# Classification of imbalanced data using machine learning algorithms to predict the risk of renal graft failures in Ethiopia

**DOI:** 10.1186/s12911-023-02185-5

**Published:** 2023-05-22

**Authors:** Getahun Mulugeta, Temesgen Zewotir, Awoke Seyoum Tegegne, Leja Hamza Juhar, Mahteme Bekele Muleta

**Affiliations:** 1 Department of Statistics, Bahir Dar University, Bahir Dar, Ethiopia; 2 School of Mathematics, Statistics, and Computer Science, KwaZulu-Natal University, Durban, South Africa; 3 St. Paul’s Hospital Millennium Medical College, Addis Ababa, Ethiopia

**Keywords:** Renal transplantation, Graft failure, Imbalanced Data, Tree-based ensembles, Stacking ensemble, Probabilistic models

## Abstract

**Introduction:**

The prevalence of end-stage renal disease has raised the need for renal replacement therapy over recent decades. Even though a kidney transplant offers an improved quality of life and lower cost of care than dialysis, graft failure is possible after transplantation. Hence, this study aimed to predict the risk of graft failure among post-transplant recipients in Ethiopia using the selected machine learning prediction models.

**Methodology:**

The data was extracted from the retrospective cohort of kidney transplant recipients at the Ethiopian National Kidney Transplantation Center from September 2015 to February 2022. In response to the imbalanced nature of the data, we performed hyperparameter tuning, probability threshold moving, tree-based ensemble learning, stacking ensemble learning, and probability calibrations to improve the prediction results. Merit-based selected probabilistic (logistic regression, naive Bayes, and artificial neural network) and tree-based ensemble (random forest, bagged tree, and stochastic gradient boosting) models were applied. Model comparison was performed in terms of discrimination and calibration performance. The best-performing model was then used to predict the risk of graft failure.

**Results:**

A total of 278 completed cases were analyzed, with 21 graft failures and 3 events per predictor. Of these, 74.8% are male, and 25.2% are female, with a median age of 37. From the comparison of models at the individual level, the bagged tree and random forest have top and equal discrimination performance (AUC-ROC = 0.84). In contrast, the random forest has the best calibration performance (brier score = 0.045). Under testing the individual model as a meta-learner for stacking ensemble learning, the result of stochastic gradient boosting as a meta-learner has the top discrimination (AUC-ROC = 0.88) and calibration (brier score = 0.048) performance. Regarding feature importance, chronic rejection, blood urea nitrogen, number of post-transplant admissions, phosphorus level, acute rejection, and urological complications are the top predictors of graft failure.

**Conclusions:**

Bagging, boosting, and stacking, with probability calibration, are good choices for clinical risk predictions working on imbalanced data. The data-driven probability threshold is more beneficial than the natural threshold of 0.5 to improve the prediction result from imbalanced data. Integrating various techniques in a systematic framework is a smart strategy to improve prediction results from imbalanced data. It is recommended for clinical experts in kidney transplantation to use the final calibrated model as a decision support system to predict the risk of graft failure for individual patients.

## Introduction

Chronic kidney disease (CKD) often progresses to end-stage renal disease (ESRD) and kidney failure [1]. End-stage renal disease (ESRD) affects 10–15% of worldwide and 14.3% of the Ethiopian population [2]. Kidney transplantation remains the gold standard treatment option for patients with ESRD, offering superior survival and quality of life and reduced costs compared to dialysis [3]. In modern medicine, transplantation remains one of the most challenging and complex fields [3]. The development of new immunosuppressive drugs and clinical care advancements considerably improved the short-term outcomes after kidney transplantation [4]. Despite this, long-term graft survival and loss due to chronic kidney dysfunction and death with a functional graft remain the leading causes of long-term graft loss [5, 6]. Therefore, predicting the risk of long-term graft failure across the transplant cohort is essential.

Machine learning (ML) refers to several techniques for recognizing patterns based on classification models and predicting new data, which have been increasingly used for diagnosing diseases [7]. Clinical risk prediction models, which include machine learning and statistical models, are omnipresent in many medical domains aimed at predicting a clinically relevant outcome using person-level information [8]. These models help to estimate a patient’s risk of having a particular disease or experiencing an event in the future based on their current characteristics [9]. According to [10], ML-based prediction models are appropriate for predicting renal transplantation outcomes, including graft failure. Particularly, the existence of several predictors and the imbalanced nature of the data make machine learning attractive in this study. These models are powerful at predicting imbalanced and high-dimensional data over conventional statistical models. However, standard ML classifiers such as logistic regression, support vector machine, and decision trees are suitable when there is a balanced proportion of classes in the outcome of interest [9]. But most medical data sets contain “normal” samples and only a small percentage of “abnormal” samples, resulting in class imbalance problems. Binary classification problems (for example, the current case) arise when there is a large negative (majority) class compared to a positive (minority) class [11]. This imbalanced class distribution needs to be considered to end with a reliable conclusion. Techniques ranging from data-level to ensemble model-based strategies, such as data resampling, cost-sensitive learning, one-class algorithms, and ensemble modeling approaches, can address class imbalance issues [12].

Although imbalance correction through resampling, like under and over-sampling, improves the balance between true positive and true negative rates, it may result in poorly calibrated models [13]. The model may perform better by balancing the class of outcome variables, but that may not represent the actual prevalence of the minority class. According to [13], resampling the original data is not a good practice in clinical prediction models. Model calibration becomes a central performance criterion in prediction models where probability estimation is required [8]. Therefore, the choice of imbalance handling requires special consideration in clinical soft prediction models (focus on probability estimates rather than class prediction) to increase the clinical utility of the models.

As far as we know, no previous machine learning-based prediction models on renal transplant outcomes used a systematic framework to experiment with spot-check of different class imbalance handling and ensemble learning, such as stacking. Accordingly, this article aimed to develop a clinical prediction model to predict the tendency of patients toward graft failure among renal post-transplant recipients in Ethiopia using the experimentally selected imbalance handling and classification algorithms.

## Methodology

### Data source and study design

The data was extracted from the retrospective cohort of kidney transplant recipients at St. Paul’s Hospital Millennium Medical College National Kidney Transplantation Center in Ethiopia. The secondary data from the hospital records of kidney transplant recipients whose follow-up visits took between September 2015 and February 2022 were extracted. The information from the patient’s follow-up charts and medical records, including epidemiological, laboratory, and clinical histories, were taken. Table 1 provides the descriptions of those variables. About 278 kidney transplant recipients with at least three follow-up visits were included in the current study. The data is a completed case with no missing values; we crosschecked the follow-up chart and other medical records to fill in the missing value; moreover, calling the patient was another option to fill in the missing data. Since the data extraction tool is developed based on the renal post-transplant follow-up guidelines and standards, no missing variables exist.

**Table 1:** Descriptions of Variables under the Investigation

No.	Feature name (class)	Feature values
1.	Graft status (factor, outcome variable)	0 = Censored, 1 = Graft failure
2.	Recipient’s age (integer)	In years
3.	Donor’s age (integer)	In years
4.	Body mass index (numeric)	In kg/m^2^
5.	Number of post-transplant admissions (integer)	In count
6.	Duration since transplant(integer)	In months
7.	Recipient’s gender (factor)	0 = Male, 1 = Female
8.	Donor’s gender (factor)	0 = Male, 1 = Female
9.	Recipient’s religion (factor)	0 = Orthodox, 1 = Muslim, 2 = Protestant, 3 = Catholic, 4 = other
10.	Recipient’s marital status (factor)	0 = Married, 1 = Unmarried
11.	Recipient’s level of education (factor)	0 = No education, 1 = Primary, 2 = Secondary, 3 = Tertiary (diploma), 4 = degree
12.	Recipient’s employment status (factor)	0 = government employee, 1 = Private employee, 2 = No working
13.	Recipient’s residence (factor)	0 = Urban, 1 = Rural
14.	Donor-recipient relationship (factor)	0 = sibling, 1 = parent, 2 = child, 3 = spouse, 4 = relatives
15.	Place of renal allograft transplantation (factor)	0 = Locally in the center, 1 = Outside the country
16.	Post-transplant regular physical exercise (factor)	0 = No, 1 = Yes
17.	The post-transplant average intake of water/day (factor)	0 = Less than 2 litters, 1 = 2–3 litters, 2 = 3–4 litters, 3 = More than 4 litters
18.	Pre-transplant history of substance abuse (factor)	0 = No, 1 = Yes
19.	Post-transplant non-adherence (factor)	0 = No, 1 = Yes
20.	Cause of end-stage renal disease (factor)	0 = Chronic glomerulonephritis, 1 = Diabetes 2 = Hypertension, 3 = Others, 4 = Unknown
21.	Pre-transplant history of co-morbidity (factor)	0 = No, 1 = Yes
22.	Pre-transplant history of dialysis (factor)	0 = No, 1 = Yes
23.	Pre-transplant history of blood transfusion (factor)	0 = No, 1 = Yes
24.	Pre-transplant history of abdominal surgery (factor)	0 = No, 1 = Yes
25.	Family history of kidney disease (factor)	0 = No, 1 = Yes
26.	Post-transplant malignancy (factor)	0 = No, 1 = Yes
27.	Post-transplant urological complications (factor)	0 = No, 1 = Yes
28.	Post-transplant vascular complications (factor)	0 = No, 1 = Yes
29.	Post-transplant cardiovascular complication (factor)	0 = No, 1 = Yes
30.	Post-transplant infection (factor)	0 = No, 1 = Yes
31.	Post-transplant diabetes (factor)	0 = No, 1 = Yes
32.	Post-transplant hypertension (factor)	0 = No, 1 = Yes
33.	An episode of hyperacute rejection (factor)	0 = No, 1 = Yes
34.	An episode of acute rejection (factor)	0 = No, 1 = Yes
35.	An episode of chronic rejection (factor)	0 = No, 1 = Yes
36.	Post-transplant gastrointestinal problems (factor)	0 = No, 1 = Yes
37.	Post-transplant glomerulonephritis (factor)	0 = No, 1 = Yes
38.	Post-transplant delayed graft functioning (factor)	0 = No, 1 = Yes
39.	Post-transplant fluid overload (factor)	0 = No, 1 = Yes
40.	Post-transplant Covid-19 (factor)	0 = No, 1 = Yes
41.	Systolic blood pressure (integer)	In mm Hg
42.	Diastolic blood pressure (integer)	In mm Hg
43.	Body weight (numeric)	In kg
44.	White blood cell count (numeric)	In G/L
45.	Hemoglobin level (numeric)	In G/DL
46.	Platelets (integer)	In count/mL
47.	Serum creatinine level	In mg/dL
48.	Blood Urea Nitrogen level	In mg/dL
49.	Glucose level (numeric)	In mg/dL
50.	Potassium level (numeric)	In mg/dL
51.	Sodium level (numeric)	In mg/dL
52.	Calcium level (numeric)	In mg/dL
53.	Phosphorus level (numeric)	In mg/dL
54.	Tacrolimus metabolism rate (Tac_MR) (numeric)	In ng/ml × 1/mg
55.	Estimated glomerular filtration rate (eGFR) (numeric)	In mL/min/1.73m^2^

### Feature selection

**Fig. 1:**
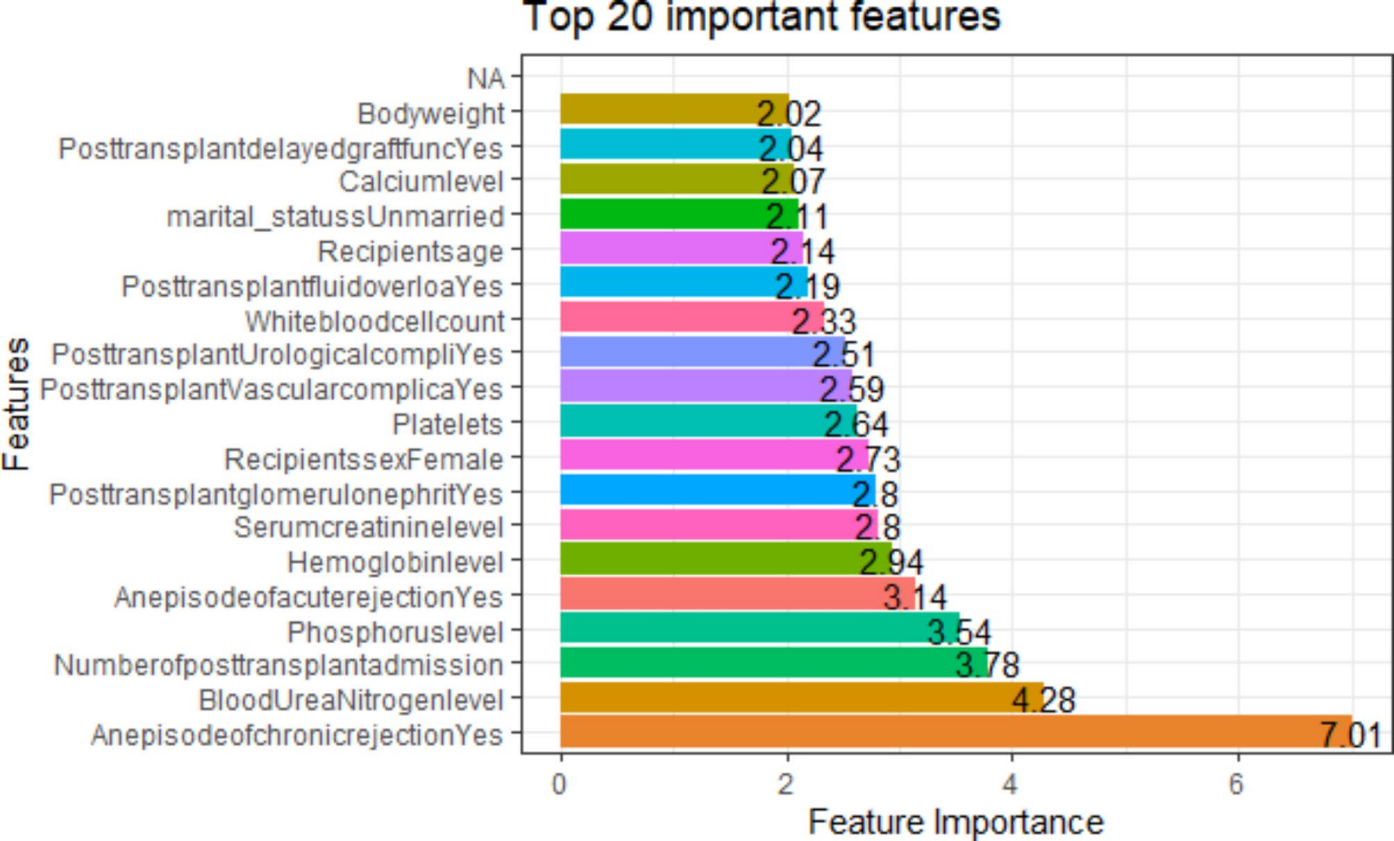
Top 20 Important Features from the Recursive Feature Elimination Method

Before developing the clinical prediction models, we performed data-driven feature selection. The goal of feature selection is to select a subset of features from the entire feature space that allows a classifier to achieve optimal performance, where it is a user-specified or adaptively parameter chosen [9]. In machine learning, the data we work with typically has many dimensions. Therefore, models built using these data are victims of the curse of dimensionality (problems associated with high dimensional datasets). Although having more information is beneficial, when the data contains duplicated or highly detailed information, the model trained on them becomes over-fitting and is likely to yield a poor-performing model [14]. We used a Recursive Feature Elimination (RFE) wrapper method (feature selection based on a specific machine learning algorithm, random forest in this case). The RFE fits the random forest model and determines how significant features are to explain the variation in the outcome variable (graft status, failed or not) using the training data set. Having determined the importance of each feature, it removes them one by one during each iteration [15]. By adjusting for multicollinearity using a correlation matrix, this variable selection process ends with the top seven predictors from the total feed of 54 variables. From the top 20 important features presented in Fig. 1, an episode of chronic rejections, blood urea nitrogen level, number of post-transplant admissions, an episode of acute rejections, post-transplant urological complication, hemoglobin level, and phosphorus level are selected as important refined features. Scaling of continuous predictors by standardization (mean centering) and identifying near-zero variance predictors (predictors with almost similar values) were performed. Accordingly, these seven refined predictor variables are used throughout the model development and testing experiments.

### Class imbalance of the outcome variable

As it has been reviewed in the introduction section, imbalanced classification refers to a classification predictive modeling problem where the number of examples in the training dataset for each class label is not balanced. From the exploratory data analysis of the response variable, it is evident that about 92.5% of the instances were grouped under the negative class (censored), while the remaining 7.5% were grouped under the positive class (graft failure). According to [16], which categorized the degree of imbalance based on the proportion of minority class as 20–40% of the data set as mild, 1–20% as moderate, and less than 1% as severe imbalance, the severity of the class imbalance for the current data lies on moderately imbalanced. Suppose we directly use this data for model training. In that case, the predictive models will undervalue the minority class (graft failure) and learn more from the majority class (censored), and we risk drawing wrong conclusions. Traditional classification algorithms have difficulty discriminating between minorities and majorities classes because the imbalance introduces a bias in favor of the majority [17]. Therefore, researchers should deal with this class imbalance problem to end up with a valid inference. Accordingly, a spot check of different imbalance handling techniques was performed to determine the appropriate and effective methods for the dataset. Decision tree-based ensemble learning, resampling, cost-sensitive learning, hyperparameter tuning, probability threshold moving, model stacking ensemble learning, and probability calibration were performed. Except for resampling and cost-sensitive learning, those experiments’ results were presented in the result section. As theoretically reviewed in the introduction, resampling is not recommended for clinical prediction, as the artificially balanced data may not represent the actual prevalence of the event of interest. Similarly, cost-sensitive were conducted by giving weight to instances based on their class distribution, but this didn’t help much. These are why the results from the two imbalance handling methods are excluded.

### Model performance evaluation metrics

The metric is the measuring stick by which all models are evaluated and compared. The wrong metric can mean choosing lousy algorithms [18]. The confusion matrix is the most commonly used metric for assessing the performance of a classification model. This metric shows how many accurate and incorrect predictions were made by a model about the actual target value [19]. It has a pool of model performance evaluation metrics; accuracy is the most commonly used. But this metric is best as there is an equal or approximately equal class proportion of the target outcome. But in severe skew in the class distributions, accuracy can become unreliable. The intuitions for classification accuracy are the leading causes of this unreliability in machine learning. Predictive modeling for classification is typically applied to the datasets when the class distribution is equal or nearly equal. Achieving a classification accuracy of 90 or even 99% during an imbalanced classification may not mean much [20]. Accordingly, the model performance evaluation metrics appropriate for imbalanced data have been used to measure the prediction performance of the machine learning algorithms. We used precision (the quality of a positive prediction), recall (or sensitivity, true positive rate), F1-score (combined information of precision and recall), and area under the curve (AUC) of receiver operating characteristic (ROC) as model evaluation metrics. These metrics are less likely to suffer from imbalanced distributions as they take class distribution into account [9]. In addition, since probability estimates are needed, the metric that evaluates the calibration of the estimated probabilities is required. Brier score is a commonly used evaluation metric that checks the goodness of a predicted probability score [21].

### The spot-check of classification algorithms

Spot-checking machine learning algorithms entails comparing various methods while modifying the hyperparameters as little as possible. The most frequently asked classification question in machine learning is ‘what is the best classification algorithm?‘ Researchers addressed the question from various perspectives, including data type. Still, the class distribution of the outcome variables has been neglected. The current study gives special emphasis to the class distribution of instances, particularly the class imbalance. Classification algorithms are chosen based on their ability to handle imbalanced data sets and directly provide probability estimates. Accordingly, logistic regression, naive Bayes, and artificial neural networks can directly provide probability estimates [22]. At the same time, decision tree-based ensemble models (random forest, bagged CART (classification and regression tree), and stochastic gradient boosting, in this case) are effective for imbalanced data sets. And their probability-like score needs to be calibrated to be interpreted as probability estimates [22].

#### Logistic regression (LR)

LR, also called the sigmoid function, is a machine-learning classification algorithm that predicts binary outcomes [11]. It predicts an event’s probability (p) and isolates positive entities from negative ones by setting a threshold boundary. It classifies as one if p > = 0.5; otherwise, it ranks as zero. In the sigmoid function, the probability value (continuous) is mapped to the discrete classes (0 and 1) [23]. Its probabilistic nature makes it appropriate for the current prediction of graft failure risk.

#### Naive Bayes (NB)

NB is a well-known classification machine learning algorithm that helps classify the data based on conditional probability values. It implements the Bayes theorem for probability computation and uses class levels represented as feature values or vectors of predictors for classification [24]. The name naive is used because it assumes the features that go into the model are independent. The NB is a good choice for classification problems requiring probability estimates [25].

#### Neural networks (NN)

Neural networks, also known as artificial neural networks (ANNs), are a subset of machine learning. They mirror how organic neurons communicate by taking their name and structure directly from the human brain [26]. An input layer, one or more hidden layers, and an output layer make up the node layers of an ANN. Each node (artificial neuron) is connected to others and has a weight and threshold that goes along with it. Any node whose output exceeds the defined threshold value is activated and provides data to the network’s uppermost layer. Otherwise, no data is transmitted to the network’s next layer. ANNs are a powerful tool in medicine for disease diagnosis and handling complex clinical conditions [27]. Due to its suitability for disease diagnosis and ability to estimate risk, this model was chosen to predict the risk of graft failure.

#### Random Forest (RF)

It is an ensemble machine learning algorithm. The central idea behind ensemble learning is that multiple weak-performing models can be combined to generate more accurate predictions. RF is the most popular machine learning algorithm, given its excellent performance across various classification and regression predictive modeling problems [28]. Based on a random selection of data samples, this algorithm creates decision trees and obtains predictions from each tree. Then, it votes/averages to determine the most viable option. Bootstrapping and aggregation (collectively known as bagging) are the two essential steps in RF. In addition to Bagging, RF selects a subset of features at each decision split to make trees independent of each other [29]. One fundamental advantage of using RF is that it eliminates the over-fitting problem since it averages or votes all predictions, canceling the biases [30]. Moreover, RF has emerged as a highly efficient and robust algorithm that can handle feature selection problems even with many variables [31]. The current study has chosen RF due to its ability to deal with over-fitting caused by small data and multiple predictors.

#### Bagged CART (TBAG)

A modified CART (classification and regression tree) algorithm, the so-called bagged CART combines bagging methods with CART to improve the performance of predictive models and reduce over-fitting [32]. Like the random forest, it is also a decision tree-based ensemble learning method. The one fundamental drawback of decision trees is that they are highly variable and unstable estimators. Bagging stands for bootstrap aggregation, an efficient approach to decreasing the prediction variance from the individual decision tree. Constructing a bagged tree is similar to the random forest algorithm, where RF uses one extra trick to keep the constituent trees less correlated while bootstrapping the training data. Instead of just sampling the training rows, it tests the features subset [33]. Similar to RF, this ensemble model was chosen because it is suitable for imbalanced data and can handle over-fitting.

#### Stochastic gradient boosting (SGB)

SGB is a modification of the gradient boosting algorithm. Gradient boosting is a greedy process that adds new decision trees to the model to fix the previous model’s error. The split points that best minimize an objective function are chosen for each decision tree using a greedy search approach [34]. As a result, trees may continually use the same attributes and even split points. So stochastic gradient boosting takes advantage of the bootstrap aggregation (bagging) technique and trains each constituent decision tree over a random sub-sample of the training dataset. This approach usually results in considerable improvements in model accuracy [35]. The model was chosen because it integrated bagging and boosting approaches to improve prediction performance.

### Hyperparameter tuning

Parameters are configuration variables learned by the machine, whereas hyperparameters are specified parameters tuned to control a machine learning algorithm’s behavior with its values set before the learning process [36]. Following the spot-checking of algorithms, the hyperparameters were adjusted using repeated cross-validation with five folds and three repetitions under the *traincontrol* function of the Caret package. We use a grid search, an exhaustive search that looks through all combinations of hyperparameters. As a result, the optimal hyperparameters of the selected classification algorithm were: naive Bayes (Laplace = 0, Usekernel = TRUE), artificial neural networks (size = 5, decay = 0.1), random forests (mtry = 2), and stochastic gradient boosting (n.trees = 50, interaction.depth = 1, shrinkage = 0.1, n.minobsinnode = 10). Logistic regression and bagged trees did not significantly benefit from the hyperparameter tuning process since they have no hyperparameters.

### Probability threshold moving

Predicting a class label is a typical aspect of classification predictive modeling [37]. However, many machine learning algorithms, like what we have mentioned above, can predict a probability of class membership, which must be interpreted before it can be mapped to a crisp class label. This grouping is commonly achieved by using a threshold of 0.5. Predicted values equal to or greater than the threshold is mapped to one class, and all other values are mapped to another category. This default threshold may perform poorly when a severe class imbalance is in a classification problem [17]. Intrinsically, a straightforward approach to improving the performance of a classifier that predicts probabilities on an imbalanced classification problem is to tune the threshold used to map probabilities to class labels [37]. Optimal thresholds cannot be calculated from the data on predictors and the actual disease status alone. Instead, the choice of threshold should reflect the harms of false positives and the benefits of true positives, which vary depending on the clinical context [38]. Moving the threshold here aims to improve the predictive models’ performance as intermediate steps. We provided the final calibrated probabilities for clinicians to determine the threshold values to discriminate between high-risk and low-risk patients by considering different clinical considerations. Testing the probability values for each positive sample makes it simple to decide on the threshold for the optimal metrics. Then, using cross-validation, the threshold with the best metrics for the training set was chosen. As a result, using the ROC curve method, the optimal threshold for this imbalanced data was 0.3.

### Stacking ensemble learning

Stacking is an ensemble machine-learning approach to determining the ideal way to integrate the forecasts from various effective machine-learning models [39]. Unlike bagging and boosting, it uses a separate model (a meta-learner) to combine the results of the base models (constituent models) [40]. Stacking-based models are mostly heterogeneous (where bagging and boosting use homogenous base models) as they tend to train different kinds (algorithmically different) of base models [40]. The meta-learner takes the outputs of base models as input and gives the prediction as to the final output. The stacking-based model can be visualized in levels and has at least two levels of the models. The first level typically trains two or more base learners. The second level might be a single meta-learner that utilizes the base model predictions as input and gives the ultimate result as output [41]. In this case, stochastic gradient boosting is used as a meta-learner. The other selected algorithms (logistic regression, naive Bayes, random forest, and bagged tree) are used as a base learner under this model stacking learning process.

### Calibrating probability estimates

Calibrated probabilities mean that the probability reflects the likelihood of actual events. Sometimes uncalibrated probabilities are biased, overconfident/under-confident [42]. Probability calibration is standard for machine learning models not trained using a probabilistic framework and for training data with a skewed distribution, like imbalanced classification tasks [43]. Most of the algorithms either predict a probability-like score or a class label. These must be coerced to produce a probability-like score. Naturally, these algorithms require their “probabilities” to be calibrated before use [44]. Hence, the probability and probability-like scores predicted by the specified algorithms were calibrated to adjust the imbalanced class. After calibrating the predicted scores from each model, an individual-level model comparison was performed before stacking the models.

### Experimental setups

**Fig. 2:**
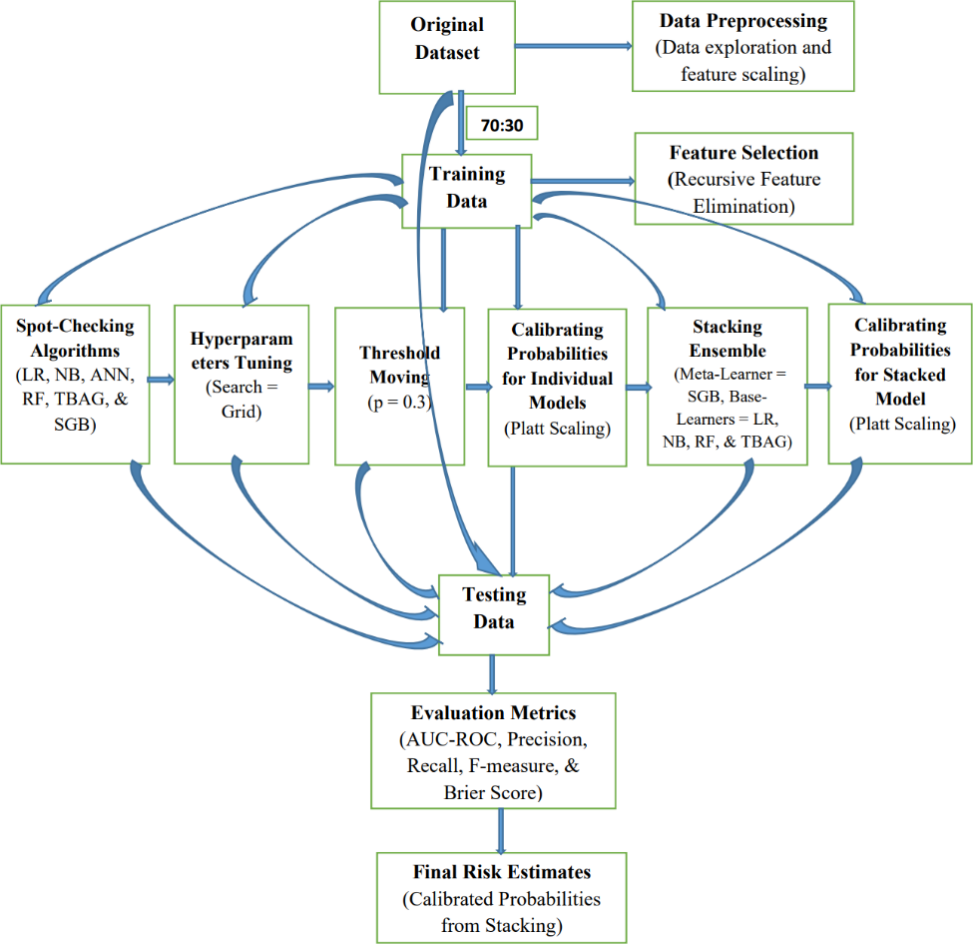
Experimental Procedures of the Study

Once the preprocessing steps, like data exploration and feature engineering, have been completed, the original data set was split into training and testing data sets with a 70:30 ratio. Due to the imbalance in the data set, a 70:30 ratio was chosen over an 80:20 to have more positive instances in the test data for better evaluation of the predictive models. The proportion of the majority to the minority class was attained using a stratified split. Accordingly, a total of 278 (21 graft failures) complete cases were split into 195 (15 graft failures) and 83 (6 graft failures) instances for training and testing sets, respectively. This training experiment was carried out via the 5-folds cross-validation method. Hence, the training data sets were divided into five folds, with equal instances in each fold. A learning algorithm was then taught on the previous four folds and tested on the current fold for each fold. The 5-fold cross-validation technique was repeated three times, with the cases ordering shuffled each time, to ensure consistent and reliable findings. The *train* function of the Caret package is used for model development with their corresponding method for the specified algorithms. The model development phase starts by spot-checking the selected algorithms without tuning to their hyperparameters. By marking the value of model evaluation metrics, the second step of hyperparameter tuning was performed using grid search; then, probability threshold moving was performed by determining the optimal threshold values using the ROC method. Improvements were recorded through the selected evaluation metrics for each phase. In the fourth phase, probability calibration was performed using the Platt scaling method (method of transforming classification outputs into a probability distribution over classes) on the individual models before applying stacking for a fair comparison of models. In the fifth step, model stacking ensemble learning was performed, and finally, calibrating the probability estimates resulting from model stacking was reported as an ultimate risk prediction result. The workflow of the experimentation is summarized in Fig. 2.

## Results

### Study characteristics

We considered completed cases of 278 kidney transplant recipients, of which 21 patients had graft failure. The number of events per predictor is three, where seven top important predictors were considered throughout the experiment. 74.8% of the patients were male, and 25.2% were female. The median age of the patients was 37 years. About 52.2% of the patients performed allograft transplantation outside the country, and 47.8% transplanted locally in the center. The results of the experiments are presented and discussed in the following sections.

### Spot-checking algorithms

**Fig. 3:**
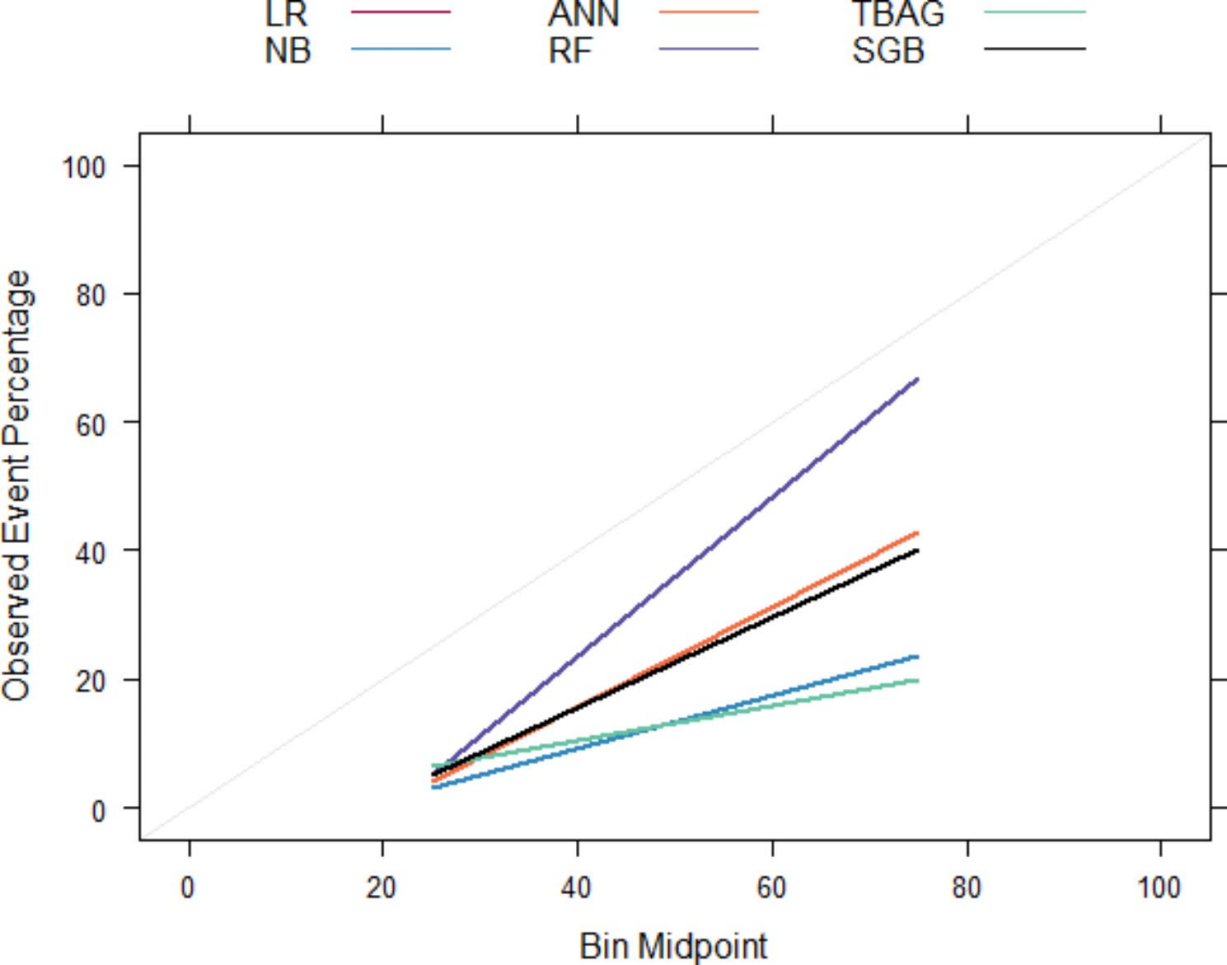
Reliability Plot for the Base Models

At the initial step of the experimentation, six selected probabilistic and tree-based ensemble algorithms were spot-checked without tuning their hyperparameters (base models). Table 2 provides model evaluation metrics’ results for each model. From the table, it is clear that those algorithms suffered from a class imbalance problem. A general overview of results in Table 2; Fig. 3 (probabilities predicted from each model were far from the actual values, 45^o^ line) shows those models performed poorly at their base level. Even if all the model performance evaluation metrics are important for class imbalance classification problems, the first (AUC-ROC) and the last (Brier Score) metrics are critical to evaluate the discrimination and calibration performance of the model, respectively. As a result, the random forest has this stage’s best discrimination performance (indicated by the largest AUC-ROC) and most calibrated probabilities (shown by the smallest brier score and reliability plot approaches to the ideal line in Fig. 3).

**Table 2:** Evaluation Metrics for Spot-checked Base Models

Algorithms	AUC-ROC	Precision	Recall	F	Brier Score
LR	0.7251082	0.4000000	0.3333333	0.3636364	0.07386198
NB	0.7424242	0.2352941	**0.6666667**	0.3478261	0.17025580
ANN	0.7619048	0.5714286	**0.6666667**	**0.6153846**	0.06708493
RF	**0.8116883**	**0.6666667**	0.3333333	0.4444444	**0.05335981**
TBAG	0.7835498	0.2000000	0.1666667	0.1818182	0.07612530
SGB	0.7240260	0.4000000	0.3333333	0.3636364	0.05739256

**Bold**: stands for selected as the best across the column.

### Hyperparameters tuning

**Fig. 4:**
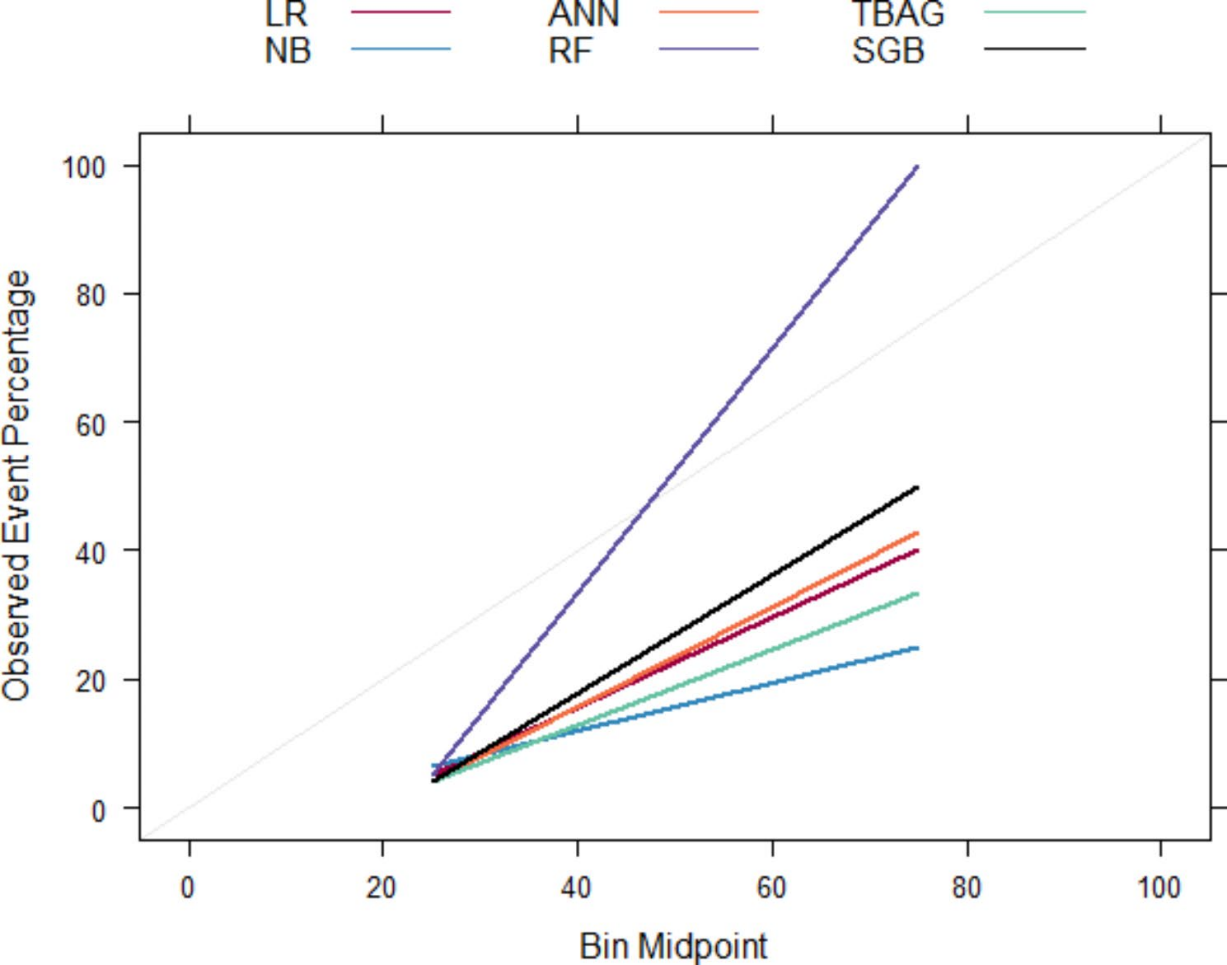
Reliability Plot for the Tuned Models

After a spot-check of algorithms, we performed hyperparameter tuning using the grid search method. Table 3 provides results for the hyperparameter tuning. The table shows that most metrics estimates improved (indicated by ↑) after tuning the base models’ hyperparameters. Figure 4 also supports the improvements from hyperparameter tuning, in which there is a shift in the calibration plot towards the ideal line compared to the calibration for the base models. The bagged tree (TBAG) discriminates best in this scenario, whereas the random forest has the most calibrated probabilities.

**Table 3:** Evaluation Metrics for the Tuned Models

Algorithms	AUC-ROC	Precision	Recall	F	Brier Score
LR	0.7251082	0.4000000	0.3333333	0.3636364	0.07386198
NB	0.755411↑	0.2500000↑	0.1666667	0.2000000	0.0916961↑
ANN	0.7489171	0.4285714	**0.5000000**	0.4615385	0.0629928↑
RF	0.821428↑	**1.0000000**↑	0.3333333	**0.5000000**↑	**0.0528066**↑
TBAG	**0.837662**↑	0.3333333↑	**0.500000**↑	0.4000000↑	0.0694168↑
SGB	0.767316↑	0.5000000↑	**0.500000**↑	**0.5000000**↑	0.0547609↑

**Bold**: column’s best estimates, ↑: shows improvement from previous approaches

### Probability threshold moving

The optimal threshold was calculated using the ROC curve method and found to be 0.3. The result of the experimentation of moving a standard threshold of 0.5 to the data-driven optimal threshold of 0.3 mainly improved recall (sensitivity, true positive rate) and F measure (harmonic mean of recall and precision). Moving the threshold reduces false negative misclassification errors, which is more costly from a clinical perspective. Like the hyperparameter tuning step, Table 4 indicates that the bagged tree has the highest discrimination performance, and the random forest produces the most calibrated probability estimates.

**Table 4:** Evaluation Metrics for Threshold Moving Experiment

Algorithms	AUC-ROC	Precision	Recall	F	Brier Score
LR	0.7251082	0.4000000	**0.6666667**↑	0.5000000↑	0.07386198
NB	0.7554113	0.2500000	0.1666667	0.2000000	0.09169611
ANN	0.7489177	0.3636364	**0.6666667**↑	0.4705882↑	0.05938057
RF	0.8214286	0.3333333	0.3333333	0.3333333	**0.05280660**
TBAG	**0.8376623**	0.3076923	**0.6666667**↑	0.4210526↑	0.06941687
SGB	0.7673160	**0.5000000**	**0.6666667**↑	**0.5714286**↑	0.05476091

**Bold**: column’s best estimates,↑: shows improvement from the previous approach

### Calibrating probability estimates before stacking the models

**Fig. 5:**
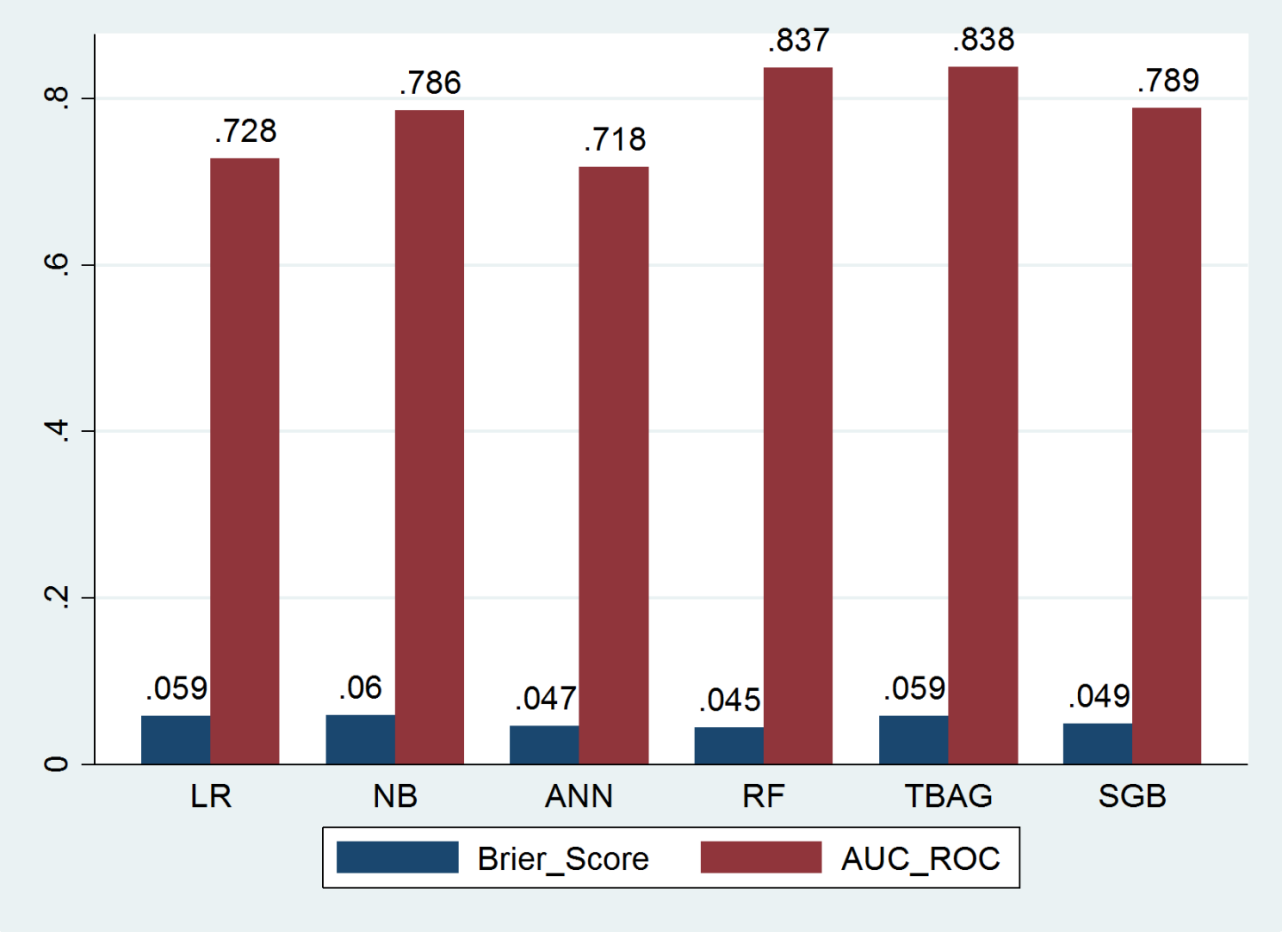
Bar Chart for the Discrimination and Calibration Performance of Individual Calibrated Models

**Fig. 6:**
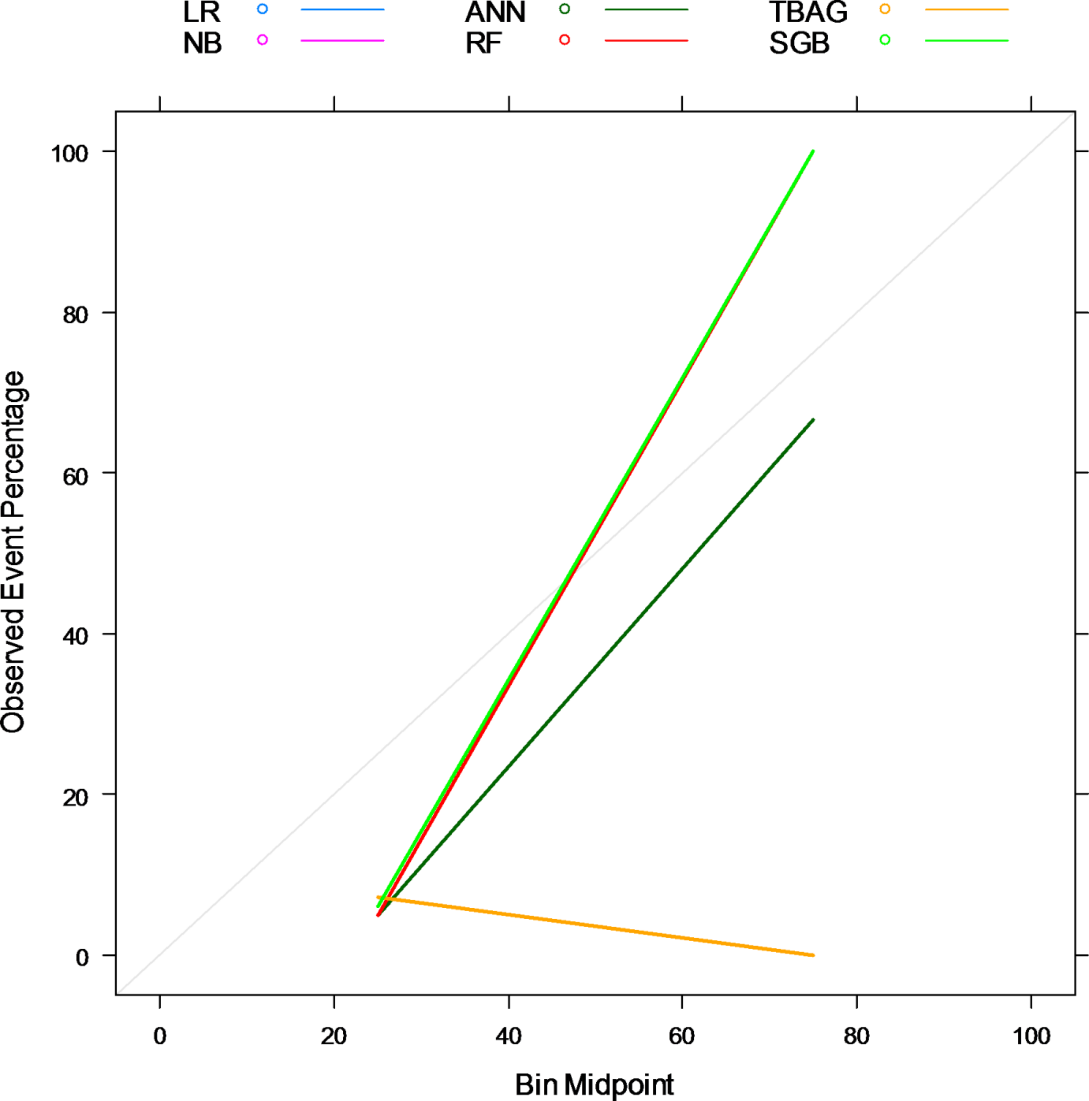
Reliability Plot for Individual Calibrated Models

In this scenario, probability estimates predicted from individual models were calibrated to compare the discrimination and calibration performance of the models. For the calibrated models, we produce a clustered bar chart (Fig. 5) and reliability plot (Fig. 6) to compare the discrimination and calibration performance of the respective model before blending (stacking) the models into a single meta-model (considering the updates from parameter tuning and threshold moving approaches). As indicated in Table 5, this calibration process improved all the probability estimates (as noted in the brier score). Unlike the threshold moving, the improvement from calibrating probabilities touches all columns of the evaluation metrics table. In this stage, the random forest and the bagged tree have almost the same discriminating performance, indicated by an approximately 84% AUC-ROC.

Regarding model calibration, even if the predicted probability from stochastic gradient boosting seems well approached to the actual values indicated by the 45^o^ straight line in Fig. 6, the random forest still has the smallest brier score in Table 5. As graphical presentations can be somewhat subjective, it is better to accept the exact Brier score figures. The lines on this plot are overlapping, and random forest and stochastic gradient boosting go together with well-calibrated lines. Therefore, the individual-level model comparison suggests that random forest is the most calibrated model to predict the risk of graft failure. Figure 6 also insights that stochastic gradient boosting more benefited from probability calibration. This result may suggest that stochastic gradient boosting can be used as a meta-learner for stacking ensemble learning to end with calibrated risk estimates.

**Table 5:** Evaluation Metrics for Calibrating Probabilities for Each Model

Algorithms	AUC-ROC	Precision	Recall	F	Brier Score
LR	0.7272727	0.4000000	0.3333333	0.3636364	0.05823289↑
NB	0.7857143↑	0.3333333↑	0.5000000↑	0.4000000↑	0.05954727↑
ANN	0.7175325	0.7500000↑	0.5000000	**0.6000000**↑	0.04688012↑
RF	0.8365801↑	**0.6666667**↑	0.3333333	0.4444444↑	**0.04498363**↑
TBAG	**0.8376623**	0.3076923	**0.6666667**	0.4210526	0.05800087↑
SGB	0.7889610↑	0.5000000	0.5000000	0.5000000	0.04895868↑

**Bold**: column’s best estimates, ↑: shows improvement from previous approaches

### Models stacking ensemble learning

The above results from the heterogeneous models were used as input for the selected meta-learner for this learning process. This meta-learner, developed by the base learner results, predicts the probabilities using a testing data set. We experimented with different combinations of base learners by rotating each model as a meta-learner. A promising result (regarding discrimination and calibration) was obtained when logistic regression, naive Bayes, random forest, and bagged tree were used as base learners. Stochastic gradient boosting was used as a meta-learner, which supports the above individual model calibration result’s suggestion. Table 6 shows a significant improvement in AUC-ROC and a slight improvement in other metrics. Since this result is used as the final risk prediction estimates, the stacked modal also needs calibration, and the result of the calibrated model is presented in Table 7.

**Table 6:** Evaluation Metrics for Models Stacking Learning

Algorithms	AUC-ROC	Precision	Recall	F	Brier Score
Stack SGB	0.8787879↑	0.4	0.6666667↑	0.5	0.05775711

**Table 7:** Evaluation Metrics for Calibrated Stacked Model

Algorithms	AUC-ROC		Precision	Recall	F	Brier Score
Stack SGB	0.8787879	0.5714286↑		0.6666667↑	0.6153846↑	0.04953749↑

### Calibrating probability estimates resulted from stacked model

**Fig. 7:**
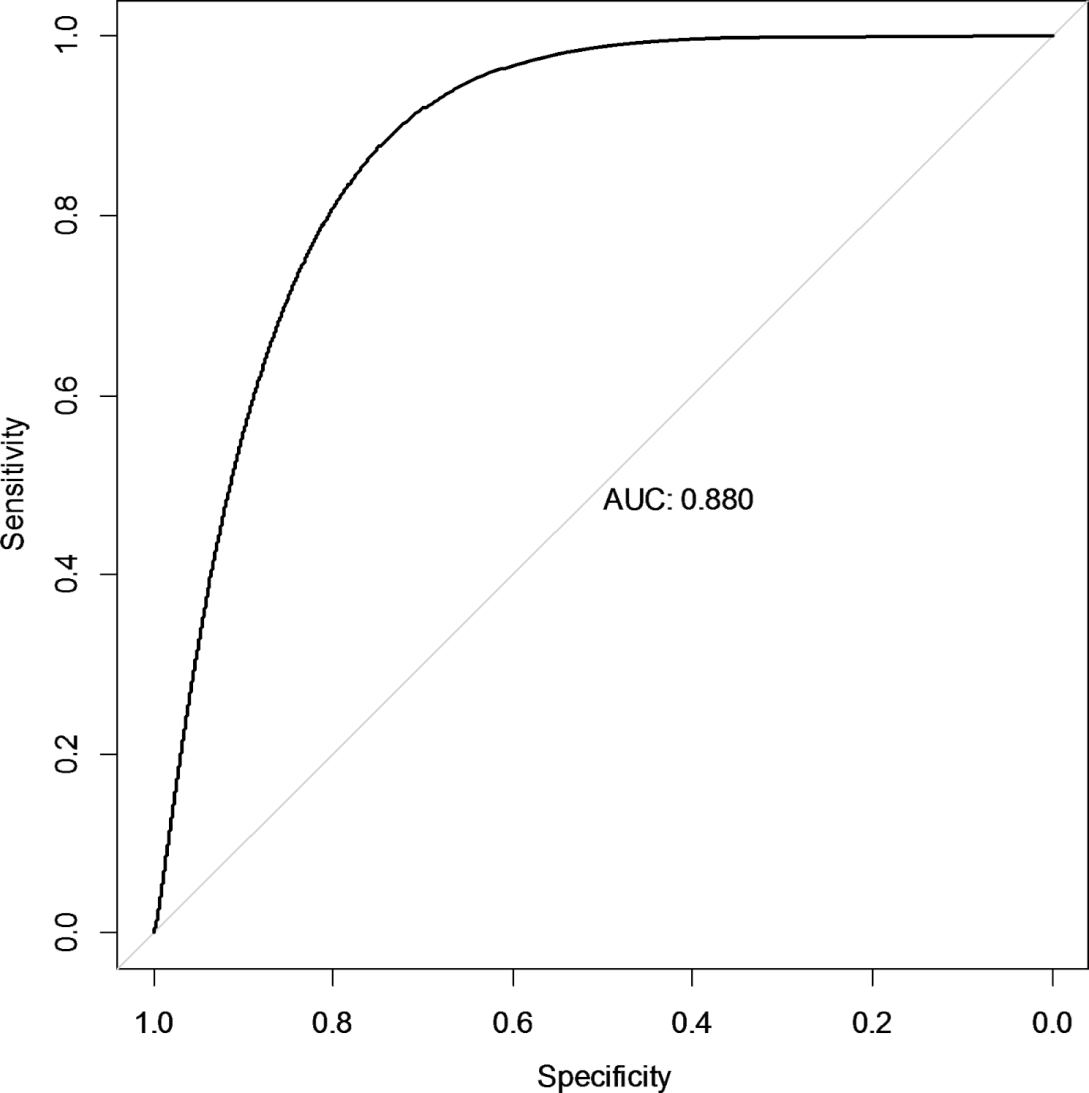
ROC Curve for the Final Calibrated Probabilities

**Fig. 8:**
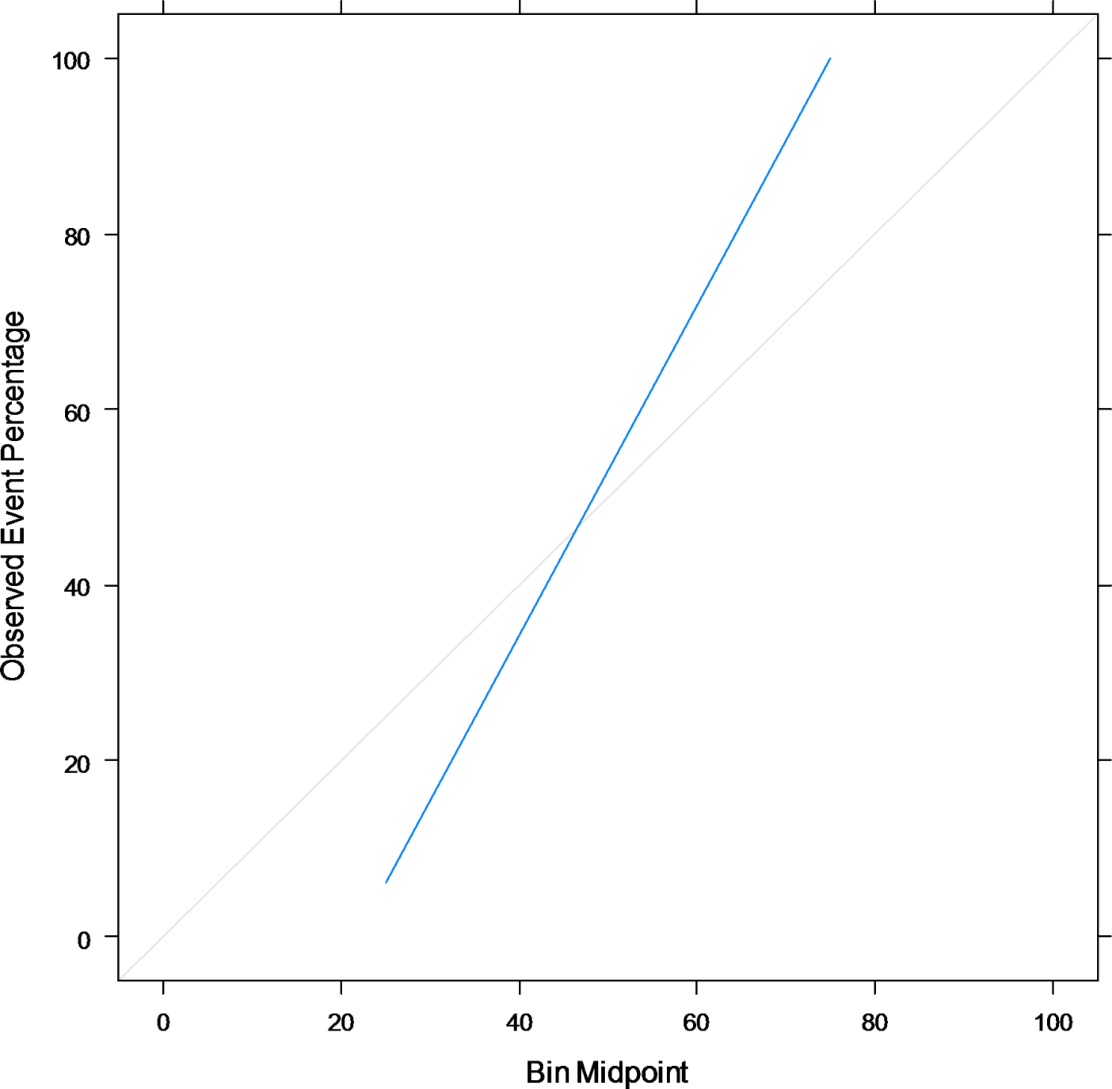
Reliability Plot for the Final Calibrated Probabilities

The final result of stacking ensemble learning has been calibrated, and the improvement of calibration has been presented in Table 7; Figs. 7 and 8. Table 7 shows that the estimates for all metrics except AUC get improved. The ROC curve and reliability plot in Figs. 7 and 8 also support this improvement. After a lot of work, we ended up with calibrated enough results. Therefore, the calibrated probabilities resulting from stacking ensemble learning are reported as absolute risks of renal graft failure for individual patients.

### Feature importance

**Fig. 9:**
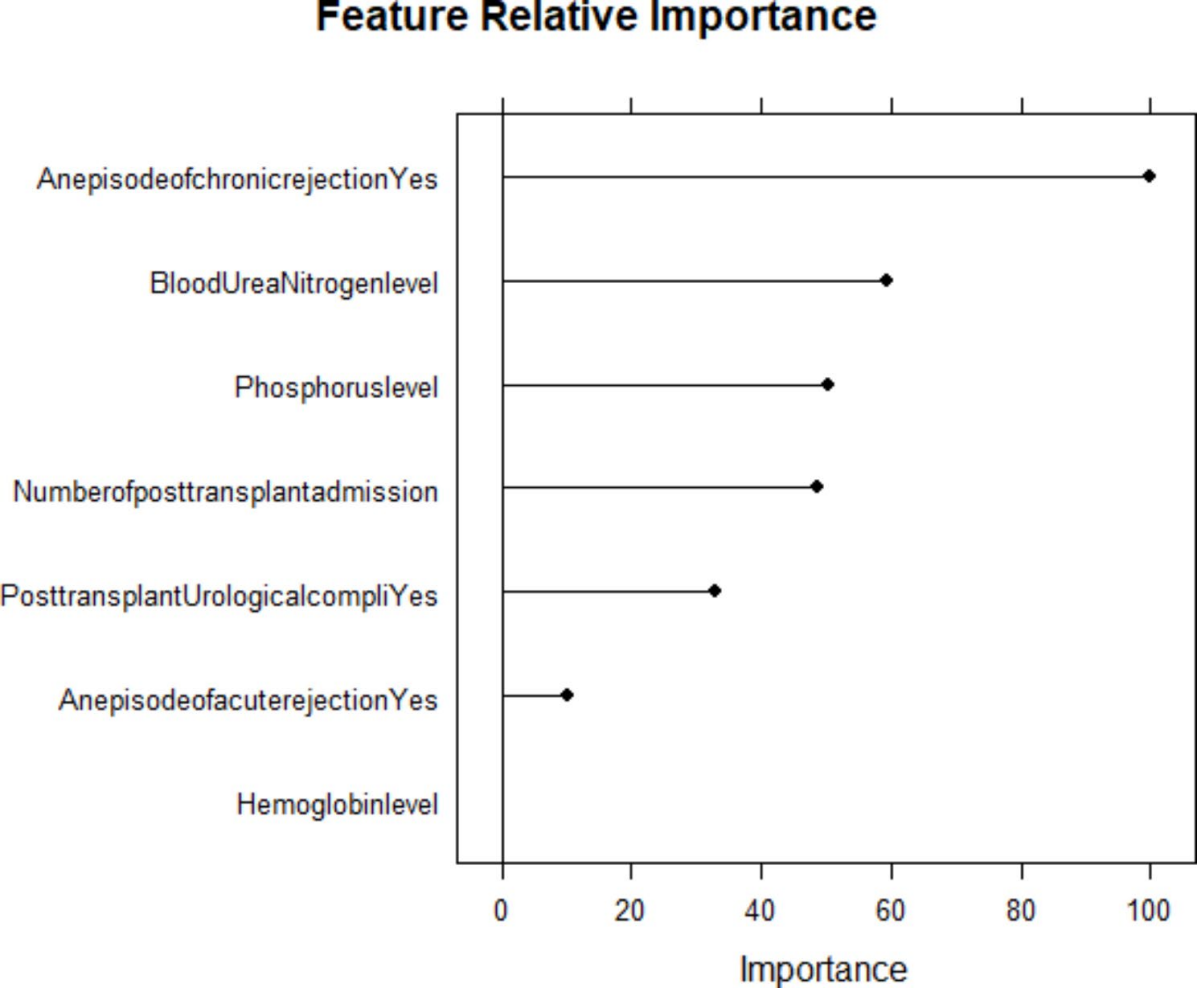
Feature’s Relative Importance to Predict Graft Failure

As mentioned in the feature selection section, we selected seven predictors to train and test the specified models. Figure 9 shows the relative importance of these chosen predictors in predicting graft failure. As a result, chronic rejections, blood urea nitrogen, the number of post-transplant admissions, phosphorus level, acute rejections, and urological complications are the top essential predictors of the risk of graft failure. Figure 9 is produced from the random forest mode, the top-performing model in terms of discrimination and calibration in the individual-level model comparison. Even if the bagged tree has equally performed with random forest and stochastic gradient boosting provides promising results in the stacking ensemble approach, due to its robustness for feature selection, random forest is used to select the important predictors.

## Discussions

This study was motivated to predict the risk of renal graft failures among kidney transplant recipients in the Ethiopian national kidney transplantation center by developing an ML-based clinical prediction model. Since the data set was imbalanced, different class imbalance handling techniques and methods of improving results were performed before reporting the final risk estimates. After a merit-based algorithms selection, extensive experiments were performed, ranging from spot-checking the base models without hyperparameter tuning to the calibrated stacking ensemble learning. Accordingly, hyperparameter tuning, probability threshold moving, calibrating individual models, stacking ensemble learning, and calibrating the stacked model demonstrate progressive improvements. Especially probability calibration and model stacking illustrate outstanding gains in the calibration and discrimination performance of the models, respectively. Even if the results are not displayed here, we demonstrate the effectiveness of resampling in improving outcomes. The result suggests that threshold moving and probability calibration well substitutes the resampling technique with a significant plurality. Therefore, these imbalance handling methods outperform resampling for prediction models where probability estimates are needed. This result is supported by [13], which states that threshold moving and probability calibration are more helpful than resampling in clinical prediction. Thus, prediction models working with imbalances should spot-check techniques to determine the effective handling method, rather than applying a single approach. It is also good practice to consider integrating different techniques for better prediction improvements, which is well established by the current study.

For clinical predictions that require risk estimates, literature [22] recommends choosing algorithms that provide probability estimates directly. But, after calibrating the predicted probabilities, tree-based ensembles (random forest, bagged tree, stochastic gradient boosting, in this case) outperform the probabilistic (logistic regression, naive Bayes, and artificial neural networks) models, which is supported by [43]. We also realized that comparing those differently configured models (probabilistic and tree-based models) without calibrating the predicted scores is not a fair practice. This is because the predicted probability-like scores from non-probabilistic models may not be interpreted as a probability (may even exceed one). The result suggests that the tree-based ensemble models benefit more from probability calibration.

The individual-level model calibration results suggest that random forest and bagged trees have top and equal discrimination performance, consistent with a previous study [45]. At the same time, the random forest has the best calibration performance, supported by a previous study [46]. The stacking ensemble, which uses stochastic gradient boosting as a meta-learner, shows significant improvements compared to the individual model’s discrimination and calibration performances, and this result is consistent with [47]. Therefore, the random forest has the top discrimination and calibration performance at the individual-level model comparison. In contrast, stochastic gradient boosting as a meta-learner has promising discrimination and calibration performance in stacking-level model comparisons. From the above successive results, we can conclude that bagging (bagged trees and random forest), boosting (stochastic gradient boosting), and stacking (blending the four models using stochastic gradient boosting as a meta-learner) are great choices for clinical predictions working on the imbalanced data. The results also showed insights that, along with discrimination, the calibration performance of the prediction model should be evaluated to enhance the clinical utility of the model. The model with the best discrimination performance may not be calibrated enough for clinical decision support.

Finally, the calibrated probability estimates predicted from the stacking ensemble were provided as the ultimate risk estimates for individual patients’ tendency to graft failure. These calibrated probability estimates enable clinical experts to determine the optimal threshold in light of different clinical considerations to stratify patients as high-risk or low-risk of graft failure. The final calibrated model from this study can be used as a decision support system in the transplant center to determine the patient-specific risk of graft failure.

Regarding feature importance, chronic rejection, blood urea nitrogen, number of post-transplant admissions, phosphorus level, acute rejection, and urological complications were the top predictors of graft failure. From a pooled analysis of results from probabilistic and tree-based models, it is evident that patients with chronic rejection, those with high blood urea nitrogen levels, those who are frequently admitted, those with high phosphorus levels, those with acute rejection, and those with urological complications are exposed to the risk of graft failure. Previous studies support these findings [48–50].

This study has limitations; applying innovative models may not guarantee itself, but data matters. The major limitations of this study are the small data and minimum event per predictor. Therefore, consideration of further research that reproduces the current approaches with large data is recommended.

## Conclusions

Tree-based ensembles, using the help of probability calibration, outperform the probabilistic models in terms of discrimination and calibration performance. Model stacking ensemble has extraordinary performance as compared to individual model performance. In a general sense, bagging, boosting, and stacking, with probability calibration, are good choices for clinical risk predictions working on imbalanced data. It is worthwhile to consider model calibration and model discrimination when assessing clinical risk predictions to enhance the clinical utility of the prediction model. Data-driven probability thresholds improve the prediction result compared to the natural threshold of 0.5. Integrating various techniques in a systematic framework is a smart strategy to improve prediction results from imbalanced data. Clinical experts for kidney transplantation may employ this final calibrated model as a decision support system with their different clinical considerations. It is also investigated that graft failure is more common in patients with chronic rejection, high blood urea nitrogen levels, frequent hospitalizations, high phosphorus levels, acute rejection, and urological complications. Accordingly, those patients need close observation and critical healthcare.

## Data Availability

The data that support the findings of this study is obtained from St. Paul’s Hospital Millennium Medical College National Kidney Transplantation Center, but restrictions apply to the availability of these data, which were used under license for the current study, and so are not publicly available. Data are, however, available from the authors upon reasonable request and with permission of St. Paul’s Hospital Millennium Medical College National Kidney Transplantation Center.

## List of abbreviations

CKD: Chronic Kidney Disease
ESRD: End Stage Renal Disease
LR: Logistic Regression
ML: Machine Learning
CART: Classification and Regression Tree
RFs: Random Forests
ROC: Receiver Operating Characteristic
AUC: Area under the Curve
SGB: Stochastic Gradient Boosting
ANN: Artificial Neural Networks
NB: Naive Bayes
TBAG: Bagged Tree
